# Priorities for tuberculosis research: a systematic review

**DOI:** 10.1016/S1473-3099(10)70201-2

**Published:** 2010-12-10

**Authors:** Jamie Rylance, Madhukar Pai, Christian Lienhardt, Paul Garner

**Affiliations:** a Malawi-Liverpool-Wellcome Trust Clinical Research Programme, Blantyre, Malawi; b International Health Group, Liverpool School of Tropical Medicine, Liverpool, UK; c Department of Epidemiology and Biostatistics, McGill University, Montreal, Canada; d Stop TB Department, WHO, Geneva, Switzerland; e Stop TB Partnership, WHO, Geneva, Switzerland

## Abstract

Reliable and relevant research can help to improve tuberculosis control worldwide. In recent years, various organisations have assessed research needs and proposed priorities for tuberculosis. We summarise existing priority statements and assess the rigour of the methods used to generate them. We found 33 documents that specifically outline priorities in tuberculosis research. The top priority areas were drug development (28 articles), diagnosis and diagnostic tests (27), epidemiology (20), health services research (16), basic research (13), and vaccine development and use (13). The most focused questions were on the treatment and prevention of multidrug-resistant tuberculosis in people co-infected with HIV. Methods used to identify these priorities were varied. Improvements can be made to ensure the process is more rigorous and transparent, and to use existing research or systematic reviews more often. WHO, Stop TB Partnership, and other organisations could adopt an incremental process of priority development, building on the existing knowledge base.

## Introduction

There are more than 9 million new cases of tuberculosis every year worldwide, and incidence is declining at a rate of less than 1% per year.^1^ Nearly 2 million people die from tuberculosis every year, and the costs and social consequences of this disease are vast. This worldwide burden of tuberculosis has stimulated much interest in research for new approaches to the management of this disease. Various organisations, individuals, and networks have tried to identify priorities to help guide and to stimulate appropriate funding. The explicit and rational setting of research priorities is integral to the research process: for allocation of resources into areas of strategic importance, to catalyse debate, and to strengthen the role of stakeholders in establishing the research agenda.^2^ Ultimately, this strategy should help to improve the allocation and monitoring of funding^3^ and the progress towards targets in tuberculosis control.^4^

Several approaches to facilitate setting of priorities for research have been described, which aim to increase transparency, objectivity, acceptability, and validity of the results. To judge the merits of competing priorities, agreed criteria are needed. Information on expected cost, existing capacity, effect of the research, and effect on the population that is expected to benefit is also needed. Several techniques have been used, including the Delphi method (iterative broad consultation with a range of experts), trend analysis and modelling (forward extrapolation of historical data on the effect of certain research), scenario discussion (assessment of current priorities on the basis of a structured discussion of the potential outcomes), and matrix approaches (that use information on cost-effectiveness and other quantifiable data on the potential effect to direct the use of restricted resources).^5^

We aimed to systematically summarise priority topics for tuberculosis research from available publications and to describe how priorities were identified. This systematic review will help to inform new initiatives for identifying and setting research priorities, such as for the recently established Research Movement of the Stop TB Partnership, which aims to increase the scope, scale, and speed of tuberculosis research and to ensure that research priorities are identified and properly funded.

## Methods

### Search strategy and selection criteria

We searched PubMed for studies published from 1998 to June 5, 2010, with the terms: (1) tuberculosis[tiab] OR mycobacter*[ti] OR (tuberculosis/epidemiology/prevention and control); (2) (research[ti] AND agend*[ti]) OR (research[ti] AND priorit*[ti]) OR (research[ti] AND need[ti]) OR (resource allocation/organisation and administration) OR (health services needs and demand) OR (research support); and (3) #1 AND #2. We also included publications cited in the documents when relevant. We contacted representatives of Stop TB Partnership and WHO to identify potentially relevant documents, especially for articles that might not have been indexed in PubMed; we also accessed information from the TB Research Movement collection. We did not use any restrictions for the language of the published studies. We excluded primary research and individual systematic reviews of specific interventions or topics. We also excluded papers that reprinted research priorities identified in previous studies.

### Data abstraction and synthesis

The search results were screened by two authors (JR and PG) independently; documents were included if there was reference to research prioritisation or research topics and specific mention of tuberculosis in the abstract. Discordant decisions were resolved by consensus.

Articles were classified into two types: consensus statements from a convened group or expert panel or reviews or commentaries on the state of tuberculosis research that mentioned priorities or agendas for future work. We noted the affiliation of authors and investigated the methods used to reach conclusions. We identified the most frequently recurring questions and areas of interest. Details were extracted and coded for each article and for each question highlighted. Data were coded according to a piloted list of summary categories to enable analysis. Data extraction was done by JR and PG, and results were directly compared for verification and entered into Microsoft Access.

## Results

1004 articles were screened and 51 were shortlisted for full-text review (figure). 18 papers were excluded (webappendix); 33 were included.^6^, ^7^, ^8^, ^9^, ^10^, ^11^, ^12^, ^13^, ^14^, ^15^, ^16^, ^17^, ^18^, ^19^, ^20^, ^21^, ^22^, ^23^, ^24^, ^25^, ^26^, ^27^, ^28^, ^29^, ^30^, ^31^, ^32^, ^33^, ^34^, ^35^, ^36^, ^37^, ^38^ 12 of 33 articles were consensus statements;^6^, ^8^, ^13^, ^20^, ^21^, ^22^, ^27^, ^28^, ^29^, ^30^, ^31^, ^32^ 11 of which were published in the past 5 years. WHO published three overviews of research priorities derived from expert consensus meetings held in 2005 with a wide range of questions. One focused on tuberculosis and HIV,^21^ one assessed tuberculosis more broadly,^22^ and the 2009 tuberculosis treatment guidelines^28^ contributed further suggestions for research arising from a series of systematic reviews. Another multidisciplinary international working group (the International Standards for TB Care steering committee) produced standards for tuberculosis care directed at healthcare providers that are applicable worldwide. As part of this process, the authors derived a set of research priorities covering clinical case management, treatment and monitoring, and operational research.^20^

**Figure:**
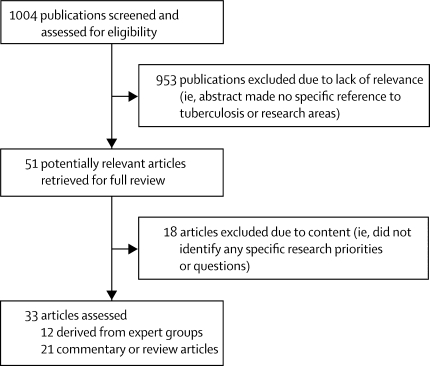
Flow chart of study selection

Three consensus statements highlighted and proposed research priorities for multidrug-resistant (MDR) tuberculosis. A consensus statement on MDR tuberculosis was published by the Stop TB Partnership's working group on MDR tuberculosis.^13^ The research subgroup of the working group on MDR tuberculosis produced a document in which they investigated the scale-up of programmatic management of MDR tuberculosis and related research priorities.^8^ A collaboration of European scientific academies made recommendations for work on MDR tuberculosis funded by the European Union.^29^ A further three consensus statements came from funding agencies, one in the USA and two from Europe. The US statement, from the National Institute of Allergy and Infectious Disease, described current and future plans for research on MDR tuberculosis funded through the National Institutes of Health.^27^ A group of authors of European Commission-funded projects produced a prioritisation programme for research in neglected infectious diseases, which made recommendations for tuberculosis research,^6^ and another article described the current and European Commission-funded tuberculosis research portfolio with recommendations for future direction of finances.^30^ The European Commission also supported another workshop on vaccine adjuvant research priorities.^31^ Lastly, a broadly drawn expert group published a paper on how to investigate the joint burden of diabetes mellitus and tuberculosis.^32^

The other 21 articles were reviews and commentaries; six focused on tuberculosis in children,^9^, ^12^, ^14^, ^25^, ^26^, ^33^ whereas the rest were not age specific. Three articles were related specifically to HIV,^7^, ^25^, ^34^ and two to MDR tuberculosis.^15^, ^35^ Seven articles summarised the broad state and priorities of tuberculosis research. Specific topics addressed were drug treatments,^11^, ^14^, ^26^, ^37^ diagnostics,^19^, ^23^, ^25^, ^36^ preventive therapy,^7^ health service limitations,^16^ funding,^17^ and design of clinical trials for tuberculosis drugs.^35^

Table 1 provides a summary of the methods used to develop the research priorities in the studies. 13 articles were derived from the questions from an expert meeting. Of these, three stated that expert advice was sought beyond the panel. Two of the consensus groups reported inclusion of representation of patients or communities. One article reported a systematic review of relevant evidence with a specific search strategy.^9^ Two groups seemed to collate data from primary research articles comprehensively, although they did not state their strategy.^7^, ^36^ Most articles (27 of 33) presented selected details from primary literature in conventional narrative review format, and gave no details of search terms, indexing databases, or inclusion and exclusion criteria.^6^, ^7^, ^10^, ^11^, ^12^, ^13^, ^15^, ^16^, ^17^, ^18^, ^19^, ^20^, ^21^, ^22^, ^23^, ^24^, ^25^, ^26^, ^27^, ^29^, ^30^, ^31^, ^32^, ^33^, ^34^, ^35^, ^38^

**Table 1:** Summary of methods used to develop research priorities for tuberculosis

	**Consensus statements (12 articles); n (%)**	**Review and commentary articles (21 articles); n (%)**	**Total (33 articles); n (%)**
Systematic reviews used	3 (25)	6 (29)	9 (27)
Search strategy specified	1 (8)	3 (14)	4 (12)
Systematic synthesis of data*	1 (8)	3 (14)	4 (12)
External advice sought beyond the panel of experts from meeting†	5 (42)	4 (19)	9 (27)
Representatives for patients involved	2 (17)	0 (0)	2 (6)
Method of question identification described	8 (67)	5 (24)	13 (39)
Method of prioritisation described	4 (33)	1 (5)	5 (15)

* Synthesis refers to a systematic analysis of primary research.

† External refers to external advice to the authors or consensus panel members.

Research areas highlighted in the studies were identified with various methods. Four of the 12 consensus statements used the opinion of expert subgroups or were derived from group discussion.^13^, ^21^, ^22^, ^30^ In three consensus papers, systematic reviews were commissioned to inform an expert group,^20^, ^28^, ^32^ and the Stop TB Partnership working group on MDR tuberculosis used established WHO guidelines to critically appraise existing publications for gaps in knowledge.^8^ The other four consensus articles gave no indication of how research areas were identified.^6^, ^27^, ^29^, ^31^ Commentary articles identified knowledge gaps by systematic review in three cases,^7^, ^9^, ^14^ and three articles discussed selected results of existing systematic reviews.^19^, ^25^, ^36^ Two reported on brainstorming exercises.^17^, ^18^ The rest of the commentary papers (13 of 21) did not specify the methods used and did not make use of systematic reviews.

Four of the consensus articles described some methods for ranking the priorities that they identified.^8^, ^13^, ^17^, ^27^ Two used meetings of experts subdivided into ad-hoc committees by research subject area; in one, the number of questions was limited to five;^13^ and in the other, participants ranked the questions they had generated by perceived importance, and refined the list by wider expert consultation.^8^ One article reported a seven-step analysis process that was used to compare research needs for the portfolio of diseases in the WHO Special Programme for Research and Training in Tropical Diseases.^17^ This paper used an analysis of current and projected burden of disease, current control limitations, research gaps, and opportunities. No further details were available from the article on how these opportunities were identified. One article used internal and external advisers to estimate the relative potential of research areas to “contribute substantially to a global public health response”,^27^ although it did not show which criteria were used to assess these relative potentials.

The number and type of research questions varied widely across the articles. Some articles provided many questions (ten suggested more than 20, of which only one group had attempted relative prioritisation)—277 question areas were identified. Research was specifically suggested for HIV-infected persons (63 questions), MDR tuberculosis (49), malnutrition (nine), and diabetes (seven). The mean number of research questions per article was 17 (range 1–78). Consensus statements had more questions on average (mean of 21 questions) than did reviews and opinion pieces (mean of 15 questions).

Research priorities were expressed differently across articles. Nine articles^9^, ^10^, ^12^, ^18^, ^20^, ^22^, ^24^, ^30^, ^33^ took into account the wide remit of tuberculosis research in general, of which three^20^, ^21^, ^22^ used expert subgroups within each area of interest. Most other articles were more focused, including five articles on MDR tuberculosis and drug development,^8^, ^13^, ^15^, ^27^, ^29^ four on HIV co-infection,^7^, ^21^, ^25^, ^34^ and two on laboratory diagnostics and vaccination.^19^, ^31^ Single articles focused individually on health system research,^16^ tuberculosis treatment guidelines,^28^ tuberculosis and diabetes mellitus,^32^ and design factors in drug trials.^35^

Most articles identified treatment with drugs, drug development, and diagnostics as areas of research priority (table 2). 17 articles identified questions relating specifically to children. With regard to HIV-infected populations, the most commonly cited questions related to antituberculous drug treatment regimens (16 articles), diagnosis (15), and epidemiology (7). The pattern for MDR tuberculosis was similar, with drug treatment (ten), diagnosis (11), and epidemiology (ten) highlighted. The panel lists the most common research areas highlighted for HIV co-infection and MDR tuberculosis. Systematic reviews were used more often for papers that had questions related to drug development, diagnostics, and epidemiology (five of nine articles that used these methods identified questions in these areas). By contrast, systematic reviews were only done in two articles that identified questions on basic science and vaccines.

**Table 2:** Number of studies identifying priority topics for tuberculosis research

		**n**
Drug development and use (7 or more articles)		28
	Studies for effectiveness of chemoprophylaxis*	9
	Optimum duration of drug treatment: new and old regimens*	9
	Development of new antituberculous drugs	7
	Pharmacokinetics of first-line drugs*	7
	Pharmacokinetics of second-line drugs*	7
	Drug interaction studies (with concomitant antiretroviral use)	7
Diagnosis and diagnostic tests (8 or more articles)		27
	New diagnostic tests for active tuberculosis*	14
	New methods for drug sensitivity testing	11
	Evaluation of diagnostic pathway for the diagnosis of active tuberculosis*	8
	Biomarkers of successful treatment (for clinical or future trial use)	8
Epidemiology and public health (5 or more articles)		20
	Accurate measurement of the global burden of tuberculosis*	8
	Identification of the role of social factors within communities on the risk of infection or transmission	5
	Effect of treatment literacy programmes on adherence and burden of disease	5
Health services research (4 or more articles)		16
	Investigation of the causes of diagnostic delay	4
	Modelling tuberculosis-associated costs or health service requirements	4
	Role of patients in case finding	4
	Best model for integration of tuberculosis and HIV services	4
	Training requirements for staff providing tuberculosis care	4
Basic science research (3 or more articles)		13
	Identification of host correlates of protection against tuberculosis	4
	Understanding latent infection and latency	4
	Understanding genetic and phenotypic markers of tuberculosis resistance	4
	Development of an animal model that can help to predict treatment duration	4
Vaccine development and use (2 or more articles)		13
	Development and trials of new tuberculosis vaccine*	8

* Questions most commonly in reference to children (four articles or more).

Panel **Common research areas about HIV co-infection and MDR tuberculosis**

**HIV**

• Optimum tuberculosis treatment using existing drugs for HIV co-infection
• Optimum duration of therapy (tuberculosis first-line drugs)
• Role of intensive case-finding in communities with high prevalence of HIV
• Studies of effectiveness of isoniazid as a preventive therapy
• Pharmacokinetic interaction studies
• Optimum role of co-trimoxazole in HIV co-infection
• Best integration of tuberculosis and HIV services
• Optimum time for initiation of antiretrovirals in tuberculosis and HIV co-infection

**MDR tuberculosis**

• New diagnostics for drug sensitivity testing
• Selection algorithms for drug sensitivity testing in existing programmes
• Use of standardised regimens for MDR tuberculosis treatment
• Efficacy studies of second-line drugs
• Safety studies of second-line drugs
• Pharmacokinetic and pharmacodynamic studies of second-line drugs
• Chemoprophylactic regimens for those in contact with MDR tuberculosis-affected individuals
• Burden of MDR tuberculosis

14 of the 33 articles were by authors who originated from academic institutions, ten were by members of WHO or Stop TB Partnership (often jointly), and nine were by authors from non-governmental organisations (five), governmental or funding agencies (three), and a science advisory body (one). 223 individuals were named as authors. Authors were most commonly affiliated with academic institutions, followed by WHO, and government staff (table 3). Representatives of funding organisations did not commonly co-author the articles (two). Patients were directly represented in two cases, both for papers on treatment guidelines. 40 individuals were listed as authors on more than one of the included articles; 12 contributed to more than two; and five contributed to more than three. Authorship of more than one article was most common for authors from universities (41%) and those affiliated with WHO and Stop TB Partnership (21%). The mean number of authors per article was nine (range 1–28); for consensus statements, the mean number of authors was 15 (range 2–28), and for reviews the mean was five (range 1–14).

**Table 3:** Author affiliations (n=223)

	**n (%)**
Academic affiliation	92 (41)
WHO or TDR or Stop TB Partnership*	46 (21)
Governmental organisation	40 (18)
Other†	45 (20)

* Author numbers from these organisations are combined.

† Authors from non-governmental organisations (18), national control programmes (nine), private sector workers (four), professional medical organisations (four), clinicians (four), funding organisations (two), medical students (one), activists for patients (two), or unknown (one). TDR=Special Programme for Research and Training in Tropical Diseases.

20 of the articles included a statement of conflicts of interest. In three articles, the author's affiliation indicated that publication would promote an organisation's interests, but no conflict of interest statement was specifically published.^10^, ^23^, ^24^ There is, however, some evidence of a recent change in practice—in articles published between 1998 and 2005, one of six articles included a statement; from 2006 to 2010, 19 of 27 did so.

## Discussion

We identified 33 research agendas for tuberculosis published between 1998 and 2010. Two clear research priorities emerged from this systematic review: the development and testing of both new drugs and treatment regimens, and new diagnostic tests for tuberculosis. These areas also dominated when focusing on the research needs in specific populations such as patients co-infected with HIV or patients with MDR tuberculosis, who are particularly affected by the limitations of current drugs and diagnostics. This finding is indicative of the inefficiencies of the currently used sputum-smear-based diagnosis in many cases and of the observation that short-course chemotherapy, despite 95% efficacy in clinical trials, has not substantially contributed to decreasing the transmission of tuberculosis in areas with high HIV burden nor been effective for patients with drug-resistant tuberculosis.^39^, ^40^

The use of epidemiology in these studies as a means to better understand the factors involved in the worldwide burden of disease and to assess the effect of case-finding was notable. The frequent inclusion of epidemiology emphasises the importance of doing studies at the population level to better target control interventions and possibly highlights a perception that accurately documenting the burden of disease might help to advocate allocation of resources for research. Epidemiological and impact-assessment studies are also necessary for monitoring and evaluation and to check if the Stop TB Partnership strategy is actually effective in controlling tuberculosis.^41^ Research into health services was also identified to be a common priority; this emphasis on operational research might be indicative of the need to optimise the availability and cost-effectiveness of techniques for improved tuberculosis control at the programme level in resource-limited settings.

Investigation of basic science questions aimed at contributing to the research and development pipeline was less commonly identified as a priority. Fundamental research on prominent fields, such as the biology of *Mycobacterium tuberculosis*, the host-pathogen interactions, and the latency and persistence patterns, is essential for the development of new diagnostic tests and drugs.^24^ Similarly, this research should support work on vaccine development, which was also relatively less prioritised than are other research areas.

According to a Treatment Action Group report on tuberculosis research and development funding, investments in tuberculosis research and development in 2007 were mostly in drug development (US$170 million, 35%), basic science ($121 million, 25%), and vaccine development ($71 million, 15%), compared with diagnostics ($41 million, 9%) and operational research ($36 million, 8%).^3^ One potential explanation for this apparent difference in allocation of funds is that, in the research topics reviewed here, the relative importance of drugs and diagnosis compared with basic and operation research is probably indicative of the difficulty of establishing clear research agendas in these areas rather than an absence of perception of priority. Basic research is mostly driven by curiosity and might therefore not benefit from fitting into specific topic areas that could be perceived as restrictive or limited. Because operational research is closely linked to programme implementation, this research falls comparatively short of funding and seems to be more difficult to prioritise in definite research agendas.

Use of systematic reviews to help establish tuberculosis research priorities is uncommon. Systematic reviews might not always be necessary; for example, because research in that area is recent. However, the use of systematic review has received growing attention in recent years and is increasingly being recognised as an important approach to the assessment of research^42^ by providing an inventory on what is known.

Although there are formal methods for prioritising research, few of the consensus groups used them. Group discussion methods tended to provide large comprehensive lists of questions without prioritisation, such as the report by WHO and the Special Programme for Research and Training in Tropical Diseases report on tuberculosis.^22^ Listing of questions might help in reaching a consensus in a large diverse group, but is less helpful in targeting strategic areas or in deciding on research funding allocation. This approach might be useful, however, for raising awareness and for funding in specific research domains. The consensus statement on short-course directly observed treatment for MDR tuberculosis, by contrast, was able to distil the list of identified topics into focused, prioritised research questions.^13^ Such an approach might be more useful in guiding scientists and funding agencies.

The pool of academics and specialists involved in these articles was relatively small and many authors contributed to more than one document. There was little representation for patients. Although we do not know what effect they would have on priorities, involvement of patients is likely to help focus research on outcomes that interest those with disease. Collaboration with patients is valuable to researchers, clinicians, and funding organisations, and is well documented in other areas.^43^ Additionally, conflicts of interest were not stated in many articles. Disclosure of conflicts of interest should be universally implemented, as has recently been adopted for expert meetings at WHO.

Although care was taken to include a comprehensive search of papers, some notable documents might have been overlooked. We studied the methods used to identify and recommend research priorities. However, this study does not answer an important question for researchers: which of the research questions is supported by high-quality evidence? We expect that by improving prioritisation methods, this question could be answered.

To identify key research questions within specific areas, various people are involved to make decisions about need and scientific opportunities. This process needs a thorough understanding of existing work and a critical analysis of existing data through systematic review where appropriate. We believe that transparent and specific processes are important in setting research priorities. Establishing these priorities would be enhanced by a structured synthesis of existing knowledge, such as that advocated by the Grading of Recommendations Assessment, Development and Evaluation (GRADE)^44^ approach.

Consensus panels understandably tend to favour discussion and critical analysis to reach agreement, but generally fail to suggest priorities. Formal methods to assess the relative merit of priority areas should be more widely adopted. This assessment would involve establishing clear criteria by which to judge the importance of the research questions, such as: the potential for progress, the public health need, and the potential effect on public health.^45^ Such an approach was adopted for research in childhood diseases.^46^ Criteria for establishing research priorities were the following: likelihood that the research question would be answerable in an ethical way; likelihood that the resulting intervention would be effective in reducing disease burden; deliverability, affordability, and sustainability of the resulting intervention; maximum potential of the intervention to reduce disease burden; and effect of disease burden reduction on equity in the population. In some approaches, a system of voting enables large groups of panel members to reach a consensus agreement. This system depends on participants being adequately informed of the existing knowledge base, such as by systematic review. Additionally, panels need to engage representatives of patients and have transparent systems for expressing potential conflicts of interest. Journals should also be consistent in requiring statements about potential conflicts of interest.

The identified research topics indicate the central role of WHO and the Stop TB Partnership in establishing guidelines and advocating for better control of tuberculosis worldwide. These organisations have recently jointly launched the TB Research Movement, which will engage many tuberculosis researchers in a collaborative strategic effort to increase the scope, scale, and speed of research to accelerate progress in worldwide control of tuberculosis.

The research areas frequently identified and summarised here should help to provide a platform for explicit development of a transparent and widely approved system for the establishment of priorities for tuberculosis research, using specific criteria and systematic reviews combined with expert opinion. Such an approach would identify knowledge gaps, inform funding organisations' decisions, and ensure that research is harmonised and effective. All these steps are crucial to improving worldwide control of tuberculosis.

## Web Extra Material

Supplementary webappendix
