# Supplementary material for: Priorities for tuberculosis research: a systematic review
Source: Lancet Infect Dis. 2010 Dec 10;10(12):889–92. doi: 10.1016/S1473-3099(10)70201-2 (PMC2992175; doi:10.1016/S1473-3099(10)70201-2)
Supplement: Supplementary webappendix [file mmc1.pdf]

THE LANCET Infectious Diseases

Supplementary webappendix

This webappendix formed part of the original submission and has been peer reviewed. We post it as supplied by the authors.

Supplement to: Rylance J, Pai M, Lienhardt C, Garner P. Priorities for tuberculosis research: a systematic review. *Lancet Infect Dis* 2010; published online Nov 2. DOI:10.1016/S1473-3099(10)70201-2.

Webappendix: Excluded articles from those initially short-listed

Author	Title	Reason for exclusion
Casenghi et al[1]	New Approaches to Filling the Gap in Tuberculosis Drug Discovery	Details barriers and solutions to organization of novel drug research. No specific TB questions.
Chakhaiyar et al[2]	Defining the mandate of tuberculosis research in a postgenomic era	Describes state of the art and current research only.
Chopra[3]	Achieving the health Millennium Development Goals for South Africa: challenges and priorities	No research questions identified; a programmatic statement on best practice in South Africa.
EASAC[4]	Drug-resistant tuberculosis: challenges, consequences and strategies for control	Comprehensively reported in a peer reviewed article.[5] Excluded to avoid duplication.
Feuer C et al[6]	Tuberculosis Research & Development: A Critical Analysis	An overview of funding arrangements and sources. No research questions or priorities identified.
Friedland G et al[7]	Tuberculosis and HIV Co-infection: Current State of Knowledge and Research Priorities	Editorial accompanying and introducing other articles only.
Ganguly et al[8]	Priorities in tuberculosis research in India	No research questions identified; a programmatic statement on current practice in India.
IUALTD[9]	Priorities for research in lung health	Not specific for tuberculosis.
Katz et al[10]	Setting the agenda: A new model for collaborative tuberculosis epidemiologic research	Describes the structure and planned research for a single organization (Tuberculosis Epidemiologic Studies Consortium).
Manley et al[11]	The Programme for Global Paediatric Research	Describes the conception of The Programme for Global Paediatric Research. No TB specific questions.
Menzieset al[12]	Meta-analysis: New Tests for the Diagnosis of Latent Tuberculosis Infection: Areas of Uncertainty and Recommendations for Research	Meta-analysis.
Narayanan et al[13]	Shifting the focus of tuberculosis research in India	Description of historical and current / ongoing research only.
NIAID[14]	NIAID Research Agenda: Multidrug-Resistant and	Comprehensively reported in a peer reviewed article.[15] Excluded to avoid

	Extensively Drug-Resistant Tuberculosis	duplication.
WHO Task Force on Priorities for Equity in Health[16]	Priorities for research to take forward the health equity policy agenda	Not specific to tuberculosis.
Rengasamy et al[17]	Respiratory protection against bioaerosols: Literature review and research needs	Not specific to tuberculosis.
Smith et al[18]	Indoor air pollution in developing countries: recommendations for research	Not specific to tuberculosis.
Walker et al[19]	The second Geneva Consensus: Recommendations for novel live TB vaccines	No research questions identified; describes current work and regulatory framework.
Young et al[20]	Ten years of research progress and what's to come	Describes existing research pipeline.

1 Casenghi M, Cole ST, Nathan CF. New approaches to filling the gap in tuberculosis drug discovery. *PLoS Med* 2007; **4:** e293.
2 Chakhaiyar P, Hasnain SE. Defining the mandate of tuberculosis research in a postgenomic era. *Med Princ Pract* 2004; **13:** 177–84.
3 Chopra M, Lawn JE, Sanders D, et al. Achieving the health Millennium Development Goals for South Africa: challenges and priorities. *Lancet* 2009; **374:** 1023–31.
4 European Academies Science Advisory Council (EASAC). Drug-resistant tuberculosis: challenges, consequences and strategies for control: EASAC, March, 2009 http://www.easac.eu/fileadmin/PDF_s/reports_statements/Drug-resistant.pdf (accessed May 21, 2010).
5 Fears R, Kaufmann S, Ter Meulen V, Zumla A. Drug-resistant tuberculosis in the European Union: opportunities and challenges for control. *Tuberculosis* 2010; **90:** 182–87.
6 Feuer C. Tuberculosis research and development: a critical analysis (2nd edn): Treatment Action Group, October 2006 http://www.stoptb.org/assets/documents/research/tbrandd2.pdf (accessed Aug 28, 2008).
7 Friedland G, Churchyard GJ, Nardell E. Tuberculosis and HIV coinfection: current state of knowledge and research priorities. *J Infect Dis* 2007; **196:** S1–3.
8 Ganguly NK, Walia K. Priorities in tuberculosis research in India. *Indian J Pediatr* 2002; **69:** S50–56.
9 International Union Against Tuberculosis and Lung Disease (IUATLD), and the International Development Research Centre (IDRC). Priorities for research in lung health. Paris, 9-11 December 1997. *Int J Tuberc Lung Dis* 1998 **2:** 1046–48.
10 Katz D, Albalak R, Wing JS, Combs V, Tuberculosis Epidemiologic Studies Consortium. Setting the agenda: a new model for collaborative tuberculosis epidemiologic research. *Tuberculosis* 2007; **87:** 1–6.
11 Manley M, Zipursky A. The programme for global paediatric research. *Arch Dis Child* 2005; **90:** 763–65.
12 Menzies D, Pai M, Comstock G. Meta-analysis: new tests for the diagnosis of latent tuberculosis infection: areas of uncertainty and recommendations for research. *Ann Intern Med* 2007; **146:** 340–54.
13 Narayanan PR, Garg R, Santha T, Kumaran PP. Shifting the focus of tuberculosis research in India. *Tuberculosis* 2003; **83:** 135–42.

14 NIAID Tuberculosis Working Group. NIAID research agenda: multidrug-resistant and extensively drug-resistant tuberculosis. National Institutes of Health; June 6, 2007 http://www.niaid.nih.gov/topics/tuberculosis/Research/Documents/mdrxdrresearchagenda.pdf (accessed Aug 28, 2008).

15 Fauci AS. Multidrug-resistant and extensively drug-resistant tuberculosis: the National Institute of Allergy and Infectious Diseases research agenda and recommendations for priority research. *J Infect Dis* 2008; **197:** 1493–98.

16 Ostlin P, Braveman P, Dachs JN, et al. Priorities for research to take forward the health equity policy agenda. *Bull World Health Organ* 2005; **83:** 948–53.

17 Rengasamy A, Zhuang Z, Berryann R. Respiratory protection against bioaerosols: literature review and research needs. *Am J Infect Control* 2004; **32:** 345–54.

18 Smith KR. Indoor air pollution in developing countries: recommendations for research. *Indoor Air* 2002; **12:** 198–207.

19 Walker KB, Brennan MJ, Ho MM, et al. The second Geneva Consensus: recommendations for novel live TB vaccines. *Vaccine*2010; **28:** 2259–70.

20 Young DB. Ten years of research progress and what's to come. *Tuberculosis* 2003; **83:** 77–81.
